# Cell death in ectopic calcification of the kidney

**DOI:** 10.1038/s41419-019-1697-8

**Published:** 2019-06-13

**Authors:** Giovanna Priante, Federica Mezzabotta, Rosalba Cristofaro, Federica Quaggio, Monica Ceol, Lisa Gianesello, Dorella Del Prete, Franca Anglani

**Affiliations:** Kidney Histomorphology and Molecular Biology Laboratory, Clinical Nephrology, Department of Medicine - DIMED, University of Padova, Padova, Italy

**Keywords:** Cell biology, Diseases

Ectopic calcification is an inappropriate biomineralization process occurring in soft tissues. Such calcifications usually consist of calcium phosphate salts, including hydroxyapatite. They sometimes contain calcium oxalates too as seen in cases of calcium nephrolithiasis.

Many in vitro and in vivo studies on the mechanisms behind calcium nephrolithiasis have revealed an association with medullary nephrocalcinosis, which involves the deposition of microscopic renal crystals in the tubular lumen (intratubular nephrocalcinosis) or the interstitium (interstitial nephrocalcinosis)^1^. The clinical, biochemical and genetic aspects of the diseases responsible for nephrocalcinosis have now been clarified in detail, but there is still a paucity of information on the specific cellular events involved in this type of calcification process. The most likely explanation for the onset of interstitial nephrocalcinosis seems to be purely physicochemical, relating to a spontaneous Ca_2_PO_4_ crystallization in the interstitium as a result of it being oversaturated with calcium and phosphate^2^. Precisely how the renal cells are involved in the response to the influx of these potentially precipitating ions is still not clear. We were the first to suggest that nephrocalcinosis could be an osteogenic cell-driven process^3^, and our earlier studies were the first to produce evidence to indicate that human renal cells can undergo a process similar to that of vascular calcification^4^. We saw a phenomenon of spontaneous biomineralization occurring in primary renal cells from a patient with medullary sponge kidney (MSK)—a clinical condition associated to nephrolithiasis and medullary nephrocalcinosis—who had a *GDNF* gene mutation. When exposed to an osteogenic medium, an immortalized proximal tubule epithelial cell line from a normal adult human kidney (HK-2) with a silenced *GDNF* expression also proved better able to produce Ca_2_PO_4_ deposits than wild-type cells. This was due to the ratio of osteonectin (a pro-osteogenic factor) to osteopontin (an anti-osteogenic factor) being regulated differently, in favor of osteonectin^4^.

The core question that remained to be answered concerned which cellular mechanisms lead to *GDNF* downregulation promoting the calcification process. We went on to investigate whether downregulated *GDNF*, which encodes the glial-derived neutrophic factor—a survival factor for many cell types, including renal cells^5^—could favor cell death.

It is common knowledge that pathological calcification is important in cell death phenomena. For instance, chondrocyte-derived apoptotic bodies might contribute to the calcification of articular cartilage^6^. In advanced carotid atherosclerotic plaques, matrix vesicle-like structures derived from vascular smooth muscle cells (VSMCs) were found to contain high levels of BAX (a pro-apoptotic member of the BCL2 family), suggesting that they may be remnants of apoptotic cells^7^. Apoptotic VSMC-derived matrix vesicle-like structures can also concentrate and crystallize calcium, triggering calcification^8^. All these findings have paved the way to the theory that the formation of apoptotic bodies could initiate the ectopic calcification of some cells under certain conditions.

In the kidney, necrotic tubular cells have been associated with renal cortical nephrocalcinosis—a rare condition that is generally the result of a severe destruction of the cortex and any condition that causes acute and prolonged shock^9^. To our knowledge, the role of cell death in the more common medullary nephrocalcinosis has yet to be explained.

We know that calcium oxalate, calcium phosphate and other crystals can induce cell death, especially in renal proximal tubule cells^10^, and that crystal size may have an influence. Sun et al.^11^ demonstrated that nano-sized crystals were the most likely to cause apoptosis, whereas micron-sized crystals caused necrosis. Lysosomes may internalize nano-sized crystals, and the resulting damage could trigger an apoptotic process. Nano-sized crystals can also pass through pores into the nucleus, where they can cause DNA cleavage into regular fragments—an important feature of apoptotic cell death. Various types of crystal can gain access to cells via a phagocytotic process, resulting in a caspase-independent cell death called necroptosis^12^.

**Fig. 1:**
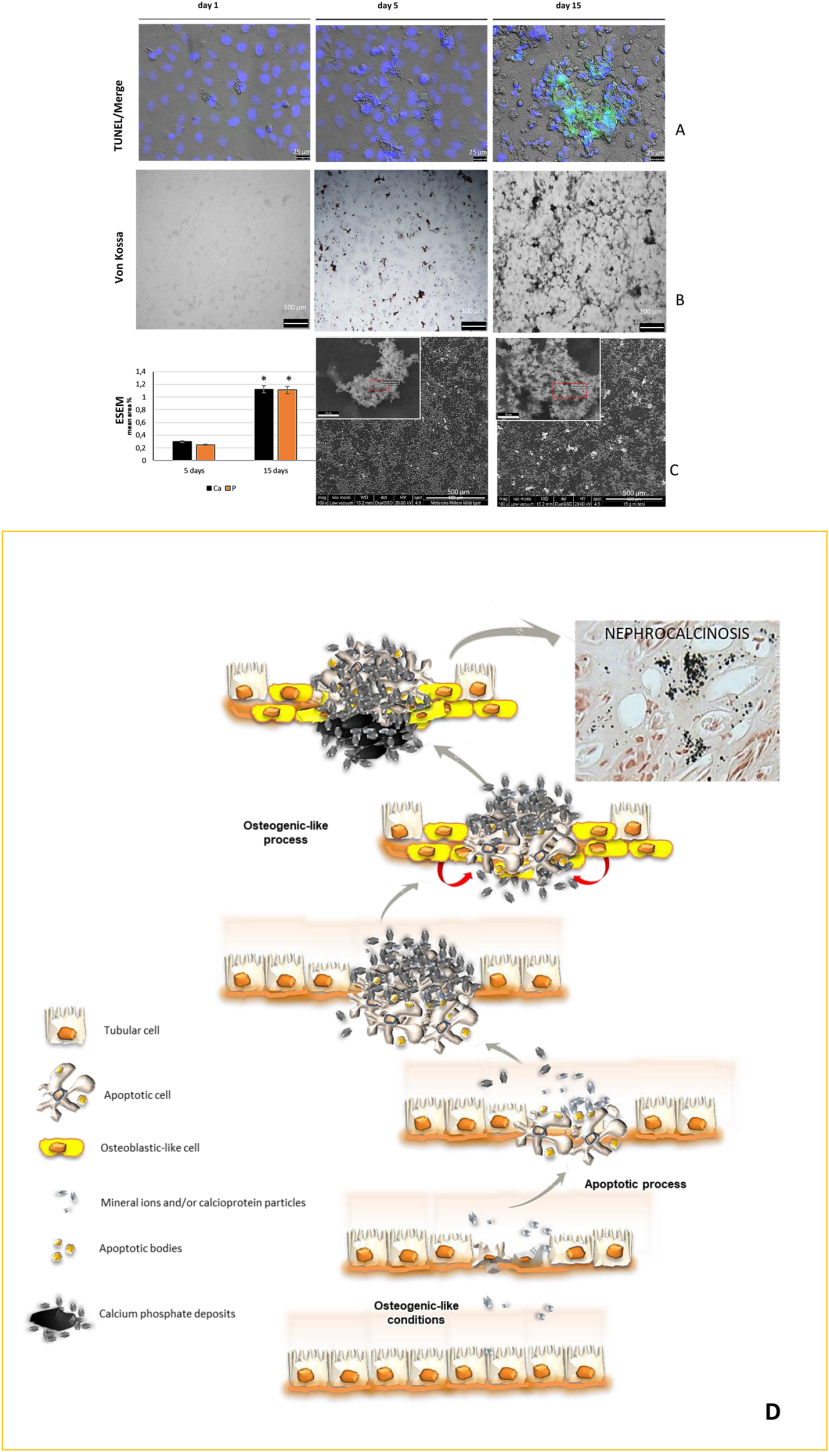
HK-2 cells in osteogenic medium show apoptosis localized in cellular aggregates of calcium phosphate. **a** Representative immunofluorescence images of DAPI and TUNEL-stained apoptotic nuclei of HK-2 cells grown in NH OsteoDiff medium for 1, 5, and 15 days. The images show DNA fragmentation localized in the nodule (bar = 25 μm). **b** von Kossa staining images of cells cultured in NH OsteoDiff medium (bar = 100 μm) show calcium phosphate deposition in multilayered cell nodules. **c** ESEM analysis. Semi-quantitative measure of the composition of the inclusions and ESEM images of selected areas of cells cultured in NH OsteoDiff medium (bar = 500 μm and 10 μm up square) (17). **d** Sequence of events for the onset of nephrocalcinosis suggested by the in vitro calcification process observed

We demonstrated the fundamental role of cell death in the onset of renal tubular cell calcification in two in vitro models of nephrocalcinosis. The first involved GDNF-silenced HK-2 cells cultured in an osteogenic medium, a model revealing how *GDNF* silencing triggered a caspase-independent cell death that strongly facilitated the formation of calcified nodules^13^. We know of several types of programmed cell death that do not involve any caspase activation^14^. The programmed cell death seen in our model would be classifiable as necroptosis because it had features coming somewhere in between apoptosis and necrosis. Since calcium phosphate aggregates were also found (albeit in much smaller quantities) in wild-type (WT) HK-2 cells cultured in osteogenic conditions, we replicated the previous experiments in WT HK-2 cells to clarify the relationship between biomineralization and apoptosis in normal tubular epithelial cells grown in an osteogenic medium. In our paper in *Cell Death Discovery*^15^, we demonstrated that HK-2 calcification with calcium (Ca) and phosphate (P) deposition was concentrated in multilayered cell nodules where the apoptotic process occurred, but the calcified cells showed the typical signs of caspase-dependent apoptosis. On monitoring the cells for two weeks, however, an apoptotic process began within 5 days of inducing osteogenesis, and the first small Ca and P crystals appeared. The importance of apoptosis in the process of HK-2 cell calcification was supported by the changes seen in *BCL2* and *BAX* gene expression. In the osteogenic medium, *BCL2* was less expressed than *BAX* from day 1 onward. This higher BAX/BCL2 ratio in the osteogenic medium than in the standard medium strongly suggests that apoptosis triggers HK-2 calcification before any Ca and P crystal deposition occurs. In fact, there was no sign of any Ca and P on von Kossa staining on the first day. Calcified deposits had become apparent by day 5, but only in or near areas where cells were apoptotic. This would indicate that early calcification is linked to HK-2 cell death, and that apoptotic areas provide the right milieu for this process—as in the case of vascular calcification. We hypothesized the following sequence of events for the calcification process we were seeing. Under osteogenic conditions, cells soon underwent apoptosis, and the subsequent release of apoptotic bodies allowed for mineral ions and/or calciprotein particles in the medium to accumulate. Then came an osteogenic-like process involving pro-osteogenic factors like Runx2, alkaline phosphatase and osteonectin upregulation, giving the impression that the osteogenic phenotype acquired by the surviving cells was a consequence rather than a cause of calcium phosphate deposition. The surviving renal tubular cells’ acquisition of an osteogenic-like phenotype could then help the process to continue (Fig. 1). If this sequence of events holds, we are tempted to speculate that any damage that causes a shift in the balance between cell survival and cell death toward the latter could (in a particular renal milieu) give rise to the phenomenon of nephrocalcinosis, and ultimately to kidney stones.
